# Quantifying landscape‐level methane fluxes in subarctic Finland using a multiscale approach

**DOI:** 10.1111/gcb.12975

**Published:** 2015-06-27

**Authors:** Iain. P. Hartley, Timothy. C. Hill, Thomas. J. Wade, Robert. J. Clement, John. B. Moncrieff, Ana. Prieto‐Blanco, Mathias. I. Disney, Brian. Huntley, Mathew. Williams, Nicholas. J. K. Howden, Philip. A. Wookey, Robert. Baxter

**Affiliations:** ^1^ Geography, College of Life and Environmental SciencesUniversity of ExeterExeterEX4 4RJUK; ^2^ Department of Earth and Environmental ScienceUniversity of St AndrewsSt AndrewsKY16 9ALUK; ^3^ School of GeosciencesUniversity of EdinburghEdinburghEH3 3JNUK; ^4^ Department of GeographyUniversity College LondonLondonWC1E 6BTUK; ^5^ NERC National Centre for Earth Observation (NCEO); ^6^ School of Biological and Biomedical SciencesUniversity of DurhamDurhamDH1 3LEUK; ^7^ Queen's School of EngineeringUniversity of BristolBristolBS8 1RJUK; ^8^ School of Life SciencesHeriot–Watt UniversityEdinburghEH14 4ASUK

**Keywords:** Aapa mire, Arctic, climate change, eddy covariance, methane oxidation, methanogenesis, remote sensing, static chambers

## Abstract

Quantifying landscape‐scale methane (CH_4_) fluxes from boreal and arctic regions, and determining how they are controlled, is critical for predicting the magnitude of any CH_4_ emission feedback to climate change. Furthermore, there remains uncertainty regarding the relative importance of small areas of strong methanogenic activity, vs. larger areas with net CH_4_ uptake, in controlling landscape‐level fluxes. We measured CH_4_ fluxes from multiple microtopographical subunits (sedge‐dominated lawns, interhummocks and hummocks) within an aapa mire in subarctic Finland, as well as in drier ecosystems present in the wider landscape, lichen heath and mountain birch forest. An intercomparison was carried out between fluxes measured using static chambers, up‐scaled using a high‐resolution landcover map derived from aerial photography and eddy covariance. Strong agreement was observed between the two methodologies, with emission rates greatest in lawns. CH_4_ fluxes from lawns were strongly related to seasonal fluctuations in temperature, but their floating nature meant that water‐table depth was not a key factor in controlling CH_4_ release. In contrast, chamber measurements identified net CH_4_ uptake in birch forest soils. An intercomparison between the aerial photography and satellite remote sensing demonstrated that quantifying the distribution of the key CH_4_ emitting and consuming plant communities was possible from satellite, allowing fluxes to be scaled up to a 100 km^2^ area. For the full growing season (May to October), ~ 1.1–1.4 g CH_4_ m^−2^ was released across the 100 km^2^ area. This was based on up‐scaled lawn emissions of 1.2–1.5 g CH_4_ m^−2^, vs. an up‐scaled uptake of 0.07–0.15 g CH_4_ m^−2^ by the wider landscape. Given the strong temperature sensitivity of the dominant lawn fluxes, and the fact that lawns are unlikely to dry out, climate warming may substantially increase CH_4_ emissions in northern Finland, and in aapa mire regions in general.

## Introduction

Wetlands release 177–284 Tg of methane (CH_4_) to the atmosphere each year and are the most important natural source of this key greenhouse gas (Ciais *et al*., 2013). To predict the potential magnitude of any feedback to climate change, it is essential that we improve our understanding of geographical variation in wetland CH_4_ fluxes, and that we determine how fluxes are controlled by key environmental variables (Gedney *et al*., 2004; Shindell *et al*., 2004). Although mapping wetland extent is challenging, it has been suggested that about half of the World's wetlands are located above 50^o^N (Mitra *et al*., 2005), occupying an area greater than two million square kilometres. However, there remains considerable uncertainty in terms of quantifying net rates of CH_4_ release from these latitudes, with fluxes calculated for boreal and arctic regions ranging from 30 to 70 Tg year^−1^ (McGuire *et al*., 2009; Ciais *et al*., 2013).

With climate change occurring most rapidly at high northern latitudes, there is the potential for CH_4_ fluxes to change substantially in the coming decades (Sitch *et al*., 2007; Koven *et al*., 2011; Zhu *et al*., 2011). Thus, it is important to determine how CH_4_ emission rates are controlled by key environmental variables in order to allow predictions of future flux rates to be made. However, the environmental control of CH_4_ fluxes is complicated, resulting in high temporal and spatial variability in fluxes. The anaerobic conditions caused by waterlogging in wetlands are essential for methanogenesis (Le Mer & Roger, 2001), but the actual magnitude of fluxes can depend strongly on plant community composition, temperature and water table depth (Olefeldt *et al*., 2013). Furthermore, in free draining, oxygenated soils, net CH_4_ uptake generally occurs (Dutaur & Verchot, 2007), as methanotrophic bacteria utilize CH_4_ as their energy source, and ultimately release the less powerful greenhouse gas, carbon dioxide. The relative role of net CH_4_ release from wetlands vs. net CH_4_ uptake in drier ecosystems in controlling landscape‐level fluxes remains poorly understood (Hendriks *et al*., 2010; Parmentier *et al*., 2011).

In Finland, nearly one‐third of the entire land surface is classified as being wetland (Turunen *et al*., 2002), and thus, rates of CH_4_ release may be considerable. The dominant wetland system in the northern boreal and subarctic regions, north of 63^o^, is the aapa mire, with this type of mire also extensive in Canada and in western and southern Siberia (Turunen *et al*., 2002). Aapa mires are considered to be mainly minerotrophic systems, containing wet, graminoid‐dominated lawns, separated by ‘ribbons’ of hummocky peat. The complex microtopography, and associated high plant community diversity, represents a challenge for quantifying CH_4_ flux rates at the wetland and landscape scale (Bridgham *et al*., 2013). Because mire subunits may be only a few metres in diameter, chamber measurements are the only suitable option for quantifying fluxes in the different plant communities. This information is essential for upscaling fluxes beyond the measurement area (Christensen *et al*., 2007; Parmentier *et al*., 2011). However, chamber measurements are associated with disturbance, and fluxes may be sensitive to measurement artefacts, such as pressure changes within the chamber (Denmead, 2008), while there are also uncertainties in upscaling vegetation community distribution unless there is very detailed remote sensing (Bridgham *et al*., 2013). Therefore, intercomparison of chamber fluxes with an independent technique such as eddy covariance can help determine the confidence that can be placed in the upscaling of chamber measurements. Furthermore, the high temporal resolution associated with eddy covariance also provides additional information on diurnal and seasonal fluctuations in CH_4_ flux rates.

Combining flux measurements made in different plant communities with the monitoring of soil temperature and moisture, and water‐table depth, make it possible to determine how CH_4_ fluxes are controlled. These data can be used to develop empirical models to calculate seasonal CH_4_ budgets for the different plant communities that characterize different hydrological conditions (Hendriks *et al*., 2010). Furthermore, by measuring fluxes in contrasting plant communities, it becomes possible to determine the relative roles of CH_4_ release from wet areas vs. CH_4_ uptake in drier ecosystems, in controlling large‐scale CH_4_ fluxes (Dutaur & Verchot, 2007; Curry, 2009); by combining flux measurements made across hydrological gradients with detailed remote sensing data, it is possible to upscale and calculate landscape‐level fluxes (Parmentier *et al*., 2011; Sturtevant & Oechel, 2013).

In our study, we took a multiscale approach, combining small‐scale chamber flux measurements with eddy covariance, aircraft‐based aerial photography and satellite remote sensing to produce a growing season CH_4_ budget for a 100 km^2^ area of northern Finland. To our knowledge, this is the first time that all of these techniques have been combined, with upscaled chamber flux measurements based on both aerial photography and IKONOS classifications being compared directly with eddy covariance measurements to give us confidence in identifying the sources and sinks of CH_4_ at the landscape scale. By linking the flux measurements with the environmental parameters monitored, we were also able to determine how the fluxes were controlled in many of the key plant communities, and thus predict whether CH_4_ fluxes are likely to increase or decrease in coming decades, as the climate warms.

## Materials and Methods

### Study area and plant community description

The core study site (69^o^29′39′′ N 27^o^13′49′′ E, altitude ~ 250 m) was located in northern Finland, ~ 50 km south of the Kevo Subarctic Research Institute, and was monitored as part of the Arctic Biosphere Atmosphere Coupling at Multiple Scales project (ABACUS; www.geos.ed.ac.uk/abacus), a contribution to the International Polar Year. The annual mean temperature is −2 °C, with mean January and July monthly temperatures of −16 °C and 13 °C, respectively.

The area is characterized by aapa mires in low‐lying, water‐logged areas (Turunen *et al*., 2002), with the free‐draining areas being dominated by mountain birch forest (*Betula pubescens* ssp. *czerepanovii* Ehrh.). These forests are very open with an understorey dominated by dwarf shrubs (*Empetrum nigrum* ssp*. hermaphroditum* L., *Betula nana* L. and *Vaccinium vitis‐idaea* L.) and lichen sp. In the areas immediately surrounding the mires, the birch trees are generally absent, with these areas being dominated by similar species to the forest understorey, lichens and dwarf shrubs (lichen‐dominated heaths). In the broader 100 km^2^ area used for the upscaling, higher ground is dominated by subarctic/alpine tundra heath, while Scots pine (*Pinus sylvestris* L.) forest is present in some valley bottoms where there are freely draining soils.

The aapa mires themselves contain wet lawns (also referred to as flarks in the literature), separated by ribbons of hummocky ground (referred to as mire edge in the aerial photography classification). The mire lawns were dominated by floating mats of graminoids including species in the genera *Eriophorum* and *Carex*, the hummocks were dominated by the shrubs *Empetrum nigrum* ssp*. hermaphroditum* and *Rhododendron tomentosum* Harmaja, and the interhummocks were generally dominated by mosses, with *Sphagnum* species in the wettest areas around the edges of the lawns. The peat in the hummocky areas of the mire was up to 2 m deep.

### Chamber flux measurements

The chamber and eddy covariance measurements were concentrated in and around an aapa mire. A series of boardwalks were set up within the mire making it possible to move around with minimal disturbance. Ten flux collars were set up in each of the three dominant mire subunits: (1) Graminoid lawns (10 collars), (2) Hummocks (10 collars) and (3) Interhummocks (10 collars). The latter included wet *Sphagnum*‐dominated areas (4 collars) and drier areas dominated by other moss species (6 collars). Fluxes were also measured in the surrounding lichen‐dominated heath (10 collars) and mountain birch forest (10 collars). In the birch forest, lichen heath, hummocks and interhummocks, 16 cm diameter PVC collars were inserted to a depth of 5 cm, with the collars being established in September 2007, nine months before the first measurements. In the interhummocks, the rate of CH_4_ release differed strongly between wet, *Sphagnum*‐dominated interhummocks (4 collars) and all other interhummocks (6 collars). Therefore, fluxes are presented separately for these two types of interhummock (i.e. interhummock and *Sphagnum*), and this difference led to the *Sphagnum* interhummocks being included with the lawns in the mire classification.

**Figure 1:**
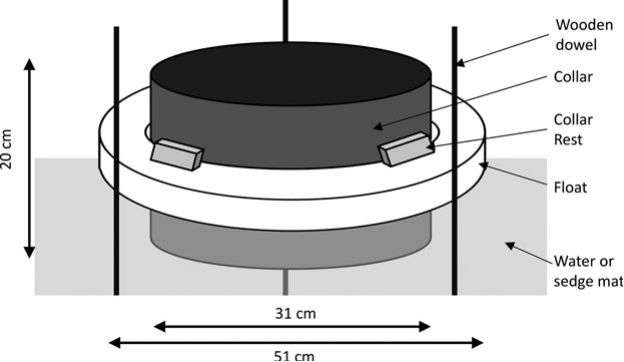
Schematic diagram showing the design of the lawn collars. The 31 cm diameter, 20 cm tall PVC collars (Collar) were inserted through a polystyrene ring (Float), with PVC blocks glued to the side of the collar (Collar rest) ensuring that the collar could not sink all the way into the water. The collar was allowed to project 5 cm below the float to ensure that a seal would be maintained if the water table fell below the level of the sedge mat. Wooden dowels were inserted in three places down to the underlying mineral sediment to reduce lateral movements.

For the lawns, collars were developed that could both float on the water surface in the wetter areas and also be gently inserted into areas with thick sedge mats. These were 20 cm deep, 31 cm diameter PVC collars that were inserted through 8 cm thick polystyrene rings (external diameter of 51 cm and an internal diameter of 31 cm), so that 5 cm of the collar projected below the float into the water or sedge mat (Fig. 1). At three points on the outside of each ring, 2 cm diameter wooden dowel was inserted through the floating vegetation mat and into the underlying sediment to limit the lateral movement of the collars. This collar design was adopted to ensure that an airtight seal would be maintained in the event of a reduction in the water level, although ultimately the floating nature of the vegetation meant that the water table never fell substantially below the level of the sedge mat. For eight of the ten collars, we found no evidence that this design promoted ebullition or that chamber deployment caused major disturbance; from these eight collars initial CH_4_ concentrations inside the chambers were below 10 ppm on 45 of the 48 measurements, and in all cases, changes in CH_4_ concentration inside the chambers were strongly linear. For the other two collars, which were located where sedge cover was reduced, the collars were less stable and attaching the chambers caused disturbance, leading to a high initial CH_4_ concentration within the chamber headspace, and nonlinear CH_4_ build‐up. For this reason, they were excluded from further analysis (*N* = 8).

All flux measurements were taken between 10:00 and 16:00, and, although it was not possible to fully randomize the order in which the collars were visited, the order was reversed between each sampling campaign. Given the height of the surface vegetation in the different plant communities, 15 cm‐tall chambers were attached to the collars using airtight rubber seals (headspace volumes: lawn chambers, 14.5–17.5 l; other chambers, 2.9–3.3 l). Gas samples were taken through a 2‐m length of PVC tubing connected to a 20‐ml syringe, allowing the operator to work remotely from the chambers further minimizing any disturbance. After flushing the syringe with chamber air, and repeated mixing of the headspace, 20 ml samples were removed from the headspace and injected into a pre‐evacuated 12 ml Exetainer^®^ (Labco Ltd., Ceredigion, UK). In the lichen heath and the birch forest, fluxes were calculated over a 1‐h closure period, with samples being collected after 0, 20 and 60 min to check for linearity of CH_4_ build‐up or uptake. For the lawns, hummocks and interhummocks, where CH_4_ emission rates were found to be higher, fluxes were measured over a 30‐min period, with samples being collected after 0, 10 and 30 min. CH_4_ concentrations in the Exetainers^®^ were measured by gas chromatography (AutoSystem XL, Perkin Elmer, Cambridgeshire, UK). CH_4_ fluxes were measured from all collars on six time points during the growing season of 2008. These periods were 11–12 June, 18–19 June, 16–17 July, 22–23 July, 25–26 August, 8–10 September.

### Environmental monitoring

After each flux measurement was completed, soil temperature and moisture within the collar were measured at 5 cm depth using a digital thermometer (E.T.I. Ltd., West Sussex, UK) and a Theta probe (ML2, Delta‐T Devices, Cambridge, UK), respectively. In the mire and surrounding lichen heath, 30 dipwells (5 cm diameter PVC tubes) were established and water‐table depth was measured manually during each set of CH_4_ measurements. Additionally, weekly measurements of water‐table depth at all locations were made to permit flux modelling. Soil temperature was also monitored continuously (thermistor probe connected to a CR10x datalogger, Campbell Scientific, Leicestershire, UK) within the wetland and birch forest.

### Eddy covariance measurements

CH_4_ fluxes were measured by eddy covariance during three campaigns: 4th to 13th June, 13th to 31st July and 19th August to 11th September. The eddy covariance tower was located within the mire towards the eastern edge (69^o^29′37.5′′N 27^o^13′48.5′′E) given that the prevailing winds at the site were from the north‐west. Fluxes were measured at a height of 3.2 m. Access to the tower was provided by a separate board‐walk to the one used to access the collars, which approached the tower from the South East. Terrain surrounding the tower was flat; according to the digital elevation map derived from stereoscopic satellite image data the standard deviation in height variation of the area within 200 m of the tower was 1.2 m. CH_4_ concentrations were measured using tunable diode laser absorption spectroscopy (TDLAS TA100, Campbell Scientific, Logan, UT, USA). The TDLAS was positioned to the south‐east and used a sample tubing of length 4 m and 0.004 m internal diameter. Three dimensional winds and air temperature were measured using a CSAT3 sonic anemometer (Campbell Scientific, Logan, UT, USA). Data were recorded at 10 Hz using a CR5000 data logger (Campbell Scientific, Logan, UT, USA). Power for the system was supplied using a generator positioned 100 m to the south‐east. Half‐hourly fluxes were calculated using the edire software package (v1.5.0.32, The University of Edinburgh). We used the Kormann & Meixner (2001) footprint model to calculate half‐hourly flux footprints with a spatial resolution of 4 by 4 m. This model is calculated within the EdiRe programme and provides an estimate of the flux footprint based on wind velocity, stability, measurement height and friction velocity (see Fig. S1 for an example of shifts in the calculated flux footprint during a 24‐h period).

### Aerial photography and vegetation classification within the study area

On 7th August 2008, aerial photographs were taken with a Canon 5D digital camera and EF 50 mm f1.4 USM lens. The camera was mounted facing downwards in an underwing pod on The University of Edinburgh's HK‐36 TTC ECO Dimona motor‐glider. A total of 270 photographs were taken from an altitude of 290 m above the ground. The images were stitched together using PTGui (version 7.8; New House Internet Services BV, Rotterdam, The Netherlands). The final composite photograph covers ~* *1000 by 3500 m and has a nominal pixel resolution of 0.05 m.

Classification of the composite aerial photograph over the field site was guided by field data and the expert opinion of scientists who had worked extensively at the site. This classification was extended to the whole of the composite aerial photograph using the image analysis software (Definiens Developer Version 7.0.6, Definiens). Following initial fragmentation, the image fragments were classified into forest and nonforest. Regions within these two classifications were then separated into finer subclasses to provide a detailed classification (see Fig. 5c). However, it was not possible to classify the drier areas of the mires (i.e. the mire edge) separately into hummocks and interhummocks, but wetter *Sphagnum*‐dominated interhummocks were recognizable and were ultimately grouped with the lawns based on their flux rates. Therefore, three vegetation classifications were identified within the mire: lawns, mire edge (hummocks and non‐*Sphagnum* interhummocks) and *Sphagnum*. Outside of the mires, it was possible to use the aerial photography to identify mountain birch forest and lichen heath. Lichen heath is included as a subcomponent under forest due to its spectral similarity with the birch forest and the fact that the IKONOS classification could not separate forest and lichen heath (see Table 2).

**Table 1:** Regression coefficients for all significant or marginally significant relationships identified between the different environmental variables and the CH_4_ fluxes measured in each vegetation community (see Table 2). The different vegetation communities are grouped by the classes identified in the aerial photography

	Temperature				Water‐table depth		Soil moisture	
	Linear		Exponential		Linear		Linear	
	*y* = *ax* + *b*		*y* = *a*.*e* ^(b.x)^		*y* = *ax* + *b*		*y* = *ax* + *b*	
	Slope	Intercept	Slope	Intercept	Slope	Intercept	Slope	Intercept
Mire: Graminoid lawn	–	–	**0.0905**	**0.0246**	–	–	–	–
Mire: *Sphagnum*	–	–	**0.148**	**0.00322**	–	–	–	–
Mire edge: Hummock	–	–	–	–	–	–	–	–
Mire edge: Interhummock	–	–	–	–	–	–	–	–
Forest: Birch forest	*−0.0732*	*−0.215*	–	–	–	–	–	–
Forest: Lichen heath	–	–	–	–	−0.0405	0.728	8.74	−4.83

The level of statistical significance is indicated (Italic: *P* < 0.1; underlined: *P* < 0.05; Bold: *P* < 0.01).

In conjunction with the EdiRE‐calculated flux footprints, the classified aerial photography was used to determine the contributions of the different vegetation communities to each of the half‐hourly CH_4_ fluxes measured by eddy covariance.

### Intercomparison between chamber and eddy covariance measurements

CH_4_ fluxes were found to vary throughout the season (see results). Therefore, the intercomparison between chambers and eddy covariance required modelling of the temporal changes in CH_4_ fluxes between chamber measurement periods. Water‐table depth was not found to be a strong predictor of rates of CH_4_ emission in this wetland, with only fluxes from the lichen heath showing a significant relationship with soil hydrological parameters (see Table 1). However, strong relationships were observed between fluxes and temperature in the key CH_4_ emitting and consuming plant communities, and thus half‐hourly CH_4_ chamber fluxes from lawn, *Sphagnum* and birch forest were modelled using empirically derived relationships with temperature (continuous measurements of soil temperature were made in both the wetland and birch forest), and lichen heath fluxes were modelled from the weekly water‐table measurements. From these modelled half‐hourly chamber measurements, we calculated the CH_4_ flux, as seen by the eddy covariance tower, using the proportional contribution of each vegetation community to the eddy covariance flux measurement in each half‐hour period (Fig. S1). To do this, the eddy covariance flux footprints were linked with the vegetation classification derived from the aerial photography to calculate the relative contribution of each land cover component for each half‐hour.

### Regional satellite data and vegetation classification

To allow for upscaling of fluxes to the landscape scale, an IKONOS stereo pair was used to create a 10 by 10 km (100 km^2^) digital elevation map (DEM) and vegetation classification map. The maps are 4 m by 4 m resolution and are approximately centred on the study site. The stereo pair was acquired on the 30th of September 2007. The DEM was georeferenced using ground control points with a vertical and horizontal accuracy of ~ 2 m. The vegetation classification was generated via supervised classification using a maximum‐likelihood classifier in ENVI version 6.3 (Exelisvis).

### Calculating CH_4_ fluxes for the 100 km^2^ area

To calculate the mean flux rates for the full 100 km^2^ area, we mapped the most important cover types (from the perspective of CH_4_ flux) using satellite data. In this way, and accounting for potential errors due to misclassification of cover types, we demonstrate a generic framework to estimate fluxes at km scale from heterogeneous mire‐dominated boreal and subarctic landscapes. The satellite data did not allow for classification of the vegetation communities in the same detail as the aerial photography, due to the lower spatial resolution (4 m, compared to 0.05 m). Only birch forests and the wettest parts of mires (graminoid lawns) were directly detectable from the satellite image, whereas hummocks/interhummocks (collectively classified as mire edge in the aerial photography) and lichen heath were not visible. It is essential to evaluate the potential impact of the less‐detailed vegetation classification in the satellite data (Bridgham *et al*., 2013). To do this, we adjusted the satellite classification based on the observed differences between the aerial photography and satellite classification within the 3.5 km^2^ area around our main study site.

Firstly, the area classified as mire in the satellite data was nearly 20% greater than the area identified as graminoid lawn in the aerial photography. To account for potential overestimation of lawn area in the satellite data compared with the fine‐scale and partially ground‐truthed aerial photography, we multiplied the area of mire detected in the satellite imagery by a factor of 0.83 to estimate the coverage of lawns.

Secondly, the aerial photography was used to estimate the area of lichen heath and hummock/interhummocks, which may have been misclassified as birch forest in the satellite data. This misclassification occurs due to the spectral similarity of these cover types, and so by (again) utilizing the fine‐scale aerial photography, we are able to account for this potential misclassification. From the aerial photography, the mire edge communities were found to occupy an area 1.32 times (with a standard error of 0.19 based on the analysis of eight equalized tiles) the area of the lawns. This ratio was used to estimate that ~ 12.5 % of the landscape may have been mire edge, and this area was subtracted from the area classified as mountain birch forest in the satellite data. Using a similar approach, we also estimated the area of lichen heath that may have been classified as birch forest in the satellite data based on the relative coverage of these two ecosystem types in the aerial photographs (35.5% of the area identified as forest was estimated to have been lichen health, after subtracting the area of mire edge).

In addition, pine forest and open tundra were present in the wider 100 km^2^ area, and no flux measurements were made in these two ecosystems. Previous studies at high latitudes have suggested that both pine forest and free‐draining tundra ecosystems are likely to show net CH_4_ uptake (Sjögersten *et al*., 2007; Matson *et al*., 2009). Therefore, as well as calculating total rates of CH_4_ uptake based on the area of birch forest within the 100 km^2^ area, and the measured chamber fluxes, we also estimated the total potential for CH_4_ uptake within the full area. To do this, we applied the rate of uptake measured in the birch forest to the full area of free‐draining ecosystems (birch forest, pine forest and tundra). This approach is appropriate because rates of CH_4_ uptake measured in birch forest in this study (~ 2 kg of CH_4_ per hectare per year) were extremely close to the median for boreal ecosystems (Dutaur & Verchot, 2007).

### Statistical analysis

For each vegetation community, linear and exponential regressions were used to evaluate the relationships between the measured environmental variables (soil temperature, soil moisture content and water‐table depth) and the chamber CH_4_ fluxes. Linear regressions were used to evaluate the strength of the relationship between upscaled chamber fluxes and eddy covariance, with daily averaged fluxes being compared. The success of the footprint model in detecting short‐term fluctuations in the contributions of the different CH_4_ emitting and consuming plant communities was evaluated by carrying out the regression analyses using both a fixed average footprint (Fig. 5d) and the dynamic footprint model (see Fig. S1).

## Results

### Chamber measurements and environmental control

**Figure 2:**
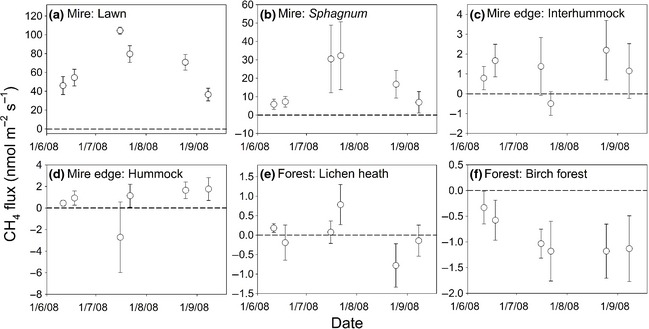
CH_4_ fluxes from the different subunits. The different vegetation communities are grouped by the classes identified in the aerial photography (Table 2). Mean fluxes ±1 SE are shown (*n* = 10, except: Mire: graminoid lawn, *n* = 8; Mire: *Sphagnum*,* n* = 4; Mire: interhummock *n* = 6). The horizontal dashed lines represent zero flux. Please note, the *y*‐axis scales differ between the different panels.

The magnitude of the CH_4_ fluxes differed between the different plant communities, with both net uptake and release being observed in contrasting communities (Fig. 2). CH_4_ emissions were greatest in the graminoid lawns. There was also net CH_4_ release from the other areas of the mire, with the *Sphagnum*‐dominated interhummocks also showing high rates of CH_4_ release. In contrast, the surrounding lichen heath was not a consistent source or sink of CH_4_, while net uptake was observed in the birch forest throughout the growing season (Fig. 2).

**Figure 3:**
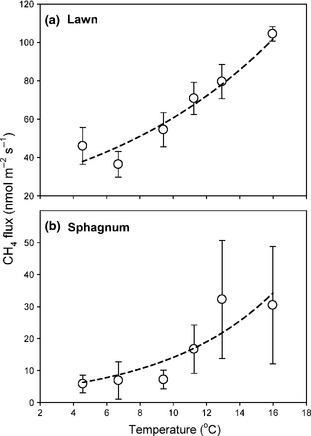
The relationship between temperature and CH_4_ emissions in (a) lawns and (b) *Sphagnum* interhummocks, the two subunits with the greatest flux rates. Mean fluxes ±1 SE are shown (lawn, *n* = 8; *Sphagnum*,* n *= 4). Exponential equations were fitted to each dataset (dashed lines), and the parameters are presented in Table 1.

CH_4_ fluxes from the lawns (*P* < 0.001, *R* ^2^ = 0.87) and *Sphagnum*‐dominated interhummocks (*P* < 0.001, *R* ^2^ = 0.78) showed strong exponential relationships with temperature (Table 1; Fig. 3). However, water‐table depth and soil moisture content were not significantly related to rates of CH_4_ emission from these areas (*P* > 0.15). In the birch forest, there was a marginally significant linear relationship between the rates of CH_4_ uptake and temperature (*P* = 0.098, *R* ^2^ = 0.42). In contrast, soil moisture content (*P* = 0.038, *R* ^2^ = 0.62) and water‐table depth (*P* = 0.015, *R* ^2^ = 0.768) were better predictors of CH_4_ fluxes from the lichen heath. The fluxes from the interhummocks and hummocks were not significantly related to any of the measured variables (Table 1).

### Eddy covariance data

**Figure 4:**
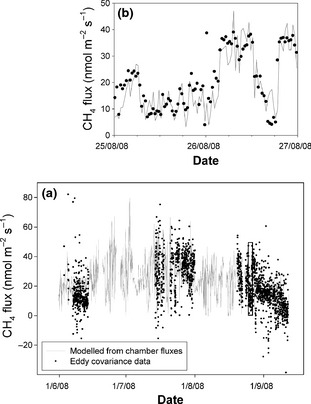
CH_4_ fluxes measured using eddy covariance and the modelled fluxes based on up‐scaling the chamber fluxes to the eddy covariance footprint. Panel (a) presents the full measurement period, while panel (b) shows a subset of the data from August (indicated by the rectangle in panel (a), demonstrating the success of the footprint model in detecting changes in the sources of the measured fluxes; the CH_4_ flux measured by eddy covariance increased and decreased as the relative proportion of lawn within the tower footprint changed, and therefore matched the modelled chamber fluxes.

**Figure 5:**
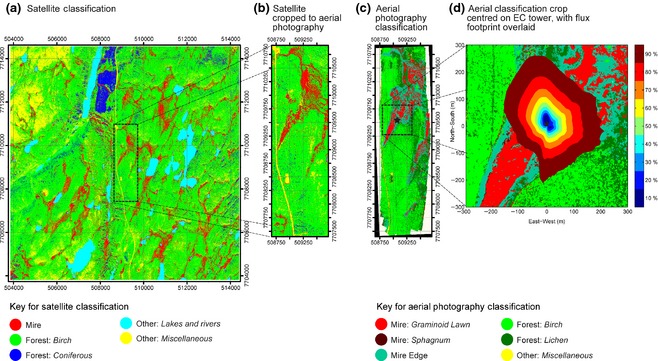
Landcover classifications based on the IKONOS satellite data (a and b) and the aerial photography (c and d). The 100 km^2^ IKONOS satellite image classification is shown in panel (a). The area corresponding to the aerial photography is highlighted with a dashed box. Panel (b) presents the crop of the satellite classification corresponding to the area covered by the aerial photography. The classification based on the aerial photography survey is shown in panel (c), with the dashed box corresponding to a 600 by 600 m area centred on the eddy covariance tower. In panel (d), the 600 by 600 m area surrounding the eddy covariance tower is shown in detail with the mean flux footprint contribution superimposed. The flux footprint presented represents the mean footprint during the eddy covariance measurement period. The colour shading of the flux footprint indicates the cumulative area contributing to the flux measurement. In both panels (c) and (d) the location of the eddy covariance tower is indicated with a star. For Panels (a), (b) and (c), coordinates are shown in UTM (zone 35N), for panel (d) the coordinate system is relative to the eddy covariance tower location (m).

CH_4_ fluxes measured by eddy covariance during the three campaigns, showed a similar seasonal pattern to the chamber fluxes measured in the main CH_4_‐emitting subunits (lawns and *Sphagnum*‐dominated interhummocks), with CH_4_ emission rates being greatest during the middle of the growing season (Fig. 4). During the first campaign in early June, eddy covariance CH_4_ fluxes remained relatively low throughout, averaging 13 nmol m^−2^ s^−1^. Rates of CH_4_ release were nearly three times as high during late July (average 32 nmol m^−2^ s^−1^) and remained relatively high at the start of the third campaign in mid‐August, but then fell rapidly in late August as temperatures declined. The flux footprint analysis indicates that eddy covariance flux measurements were, on average, derived mainly from the mire, with only a small fraction of the flux from the surrounding vegetation communities (Fig. 5d). However, there were short periods in which nonmire communities contributed substantially to the measured flux (e.g. see Fig. 4b).

### Chamber, eddy covariance intercomparison

**Figure 6:**
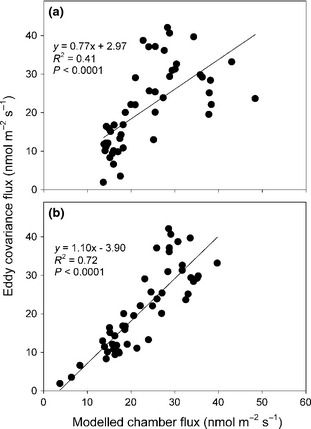
The relationship between daily averaged up‐scaled chamber fluxes and daily averaged eddy covariance fluxes. The chamber fluxes were up‐scaled both using: (a) a fixed average footprint, as shown in Fig. 5d and (b) dynamic half‐hourly eddy covariance footprints, examples of which are shown in Fig. S1.

The chamber measurements were upscaled for comparison with the eddy covariance data, based on the coverage of the different plant communities within the modelled flux footprint and empirically derived temperature–response and water table‐response functions (Table 1). A high level of agreement was observed between the upscaled chamber fluxes and the eddy covariance data (Figs 4 & 6). The two methods were in close agreement during the third campaign, while the fluxes during the first campaign appeared to be slightly higher when calculated based on chamber upscaling. On the other hand, there was also a period during the second half of July when fluxes measured by eddy covariance were slightly higher than those based on the chamber upscaling (Fig. 4). However, the strong overall agreement between these two methods, both in terms of the magnitude and temporal pattern of CH_4_ fluxes, provided strong justification for upscaling the CH_4_ fluxes to the wider 100 km^2^ area.

The intercomparison also provided a direct test of the footprint model itself. Overall, a positive correlation was also observed between the area of lawn calculated to be within the eddy covariance footprint and the flux of CH_4_ (*P* < 0.001, Fig. S2), even prior to accounting for variation in temperature. Compared with maintaining a fixed flux footprint, it was clear that dynamic footprint modelling greatly increased the agreement between the eddy covariance data and upscaled chamber fluxes (Fig. 6). Furthermore, during a period in which a temporary change in wind direction reduced the amount of mire calculated to be in the eddy covariance footprint, the CH_4_ flux declined accordingly, before increasing when the wind direction changed again ~ 6 h later (Fig. 4b). Indeed, during this period, the flux declined by near identical magnitudes in the eddy covariance data and the modelled upscaled chamber fluxes. In summary, the footprint model appeared to provide a robust estimate of the area of the key CH_4_‐emitting subunits within the tower footprint, and there was very strong agreement between the two flux measurement approaches in terms of the magnitude and temporal variation in the CH_4_ fluxes.

### Satellite data and intercomparison with aerial photography

For the ~ 1000 by 3500 m area around our study site, we compared the classifications produced by the aerial photography and the satellite data (Table 2). There was strong spatial agreement between the areas classified as graminoid lawn in the aerial photography and the areas classified as mire in the satellite data (Figs 5b & c; Figs. S3 & S4). In contrast, the hummock and interhummock areas (classified as mire edge in the aerial photography) were generally misclassified as birch forest in the satellite data (Figs 5b & c). Overall, the total area classified as mire in the satellite data was ~ 20% greater than the calculated area of graminoid lawn in the aerial photography (Table 2). Because many of the lawns were quite small features, the difference between the two classifications may have been partly caused by the coarser 4 × 4 m resolution in the satellite data. However, because the IKONOS data detected directly the area of the lawn, upscaling the chamber flux measurements based on the raw IKONOS classification actually showed good agreement with both the eddy covariance data and the chamber flux measurements upscaled using aerial photography; the fluxes based on the IKONOS classification were on average 20% higher than the fluxes calculated from the aerial photography (Fig. S5).

**Table 2:** Areal extent (% cover) of the different landscape units within the 1000 m by 3500 m area around the study site (columns 1 and 2). The results from both the aerial photography and the IKONOS satellite data are presented. Columns 3 and 4 show the areal extent of the different vegetation classes in the full 100 km^2^ area, with column 3 showing the raw classification and column 4 the adjusted classification given the potential overestimation of lawn area by using ‘mire’ (error of commission) in the raw classification. Also included is the estimated coverage of the vegetation communities that were not detected by the satellite, calculated based on relationships measured between the proportional coverage of the different vegetation types in the aerial photography (see Calculating CH_4_ fluxes for the 100 km^2^ area in Materials and Methods)

Landcover type (*Sub‐component)*	% Component Cover, (*% Sub‐component Cover*)				Comments
	For 1000 x 3500 m area around the tower		For the full 10 x 10 km^2^ area		
	Aerial Photography	IKONOS	IKONOS	Adjusted IKONOS	
Mire	11.32	13.63	11.13	9.41	Mire detectable using aerial or IKONOS images
(Graminoid Lawn)	(11.15)	(n/a)	(n/a)	(n/a)	Mire sub‐components not distinguished using IKONOS
(Sphagnum)	(0.17)	(n/a)	(n/a)	(n/a)	Mire sub‐components not distinguished using IKONOS
Mire Edge	14.74	n/a	n/a	12.42	Mire edge detected as Forest by IKONOS
(Hummocks)	(n/a)	(n/a)	(n/a)	(n/a)	Mire sub‐components not distinguished using aerial images
(Interhummocks)	(n/a)	(n/a)	(n/a)	(n/a)	Mire sub‐components not distinguished using aerial images
Forest	72.66	78.25	70.17	59.64	Forest detectable using aerial or IKONOS images
(Birch)	(46.9)	(76.5)	(65.92)	(35.75)	n/a
(Coniferous)	(0.0)	(1.8)	(4.25)	(4.25)	n/a
(Lichen Heath)	(25.8)	(n/a)	(n/a)	(19.64)	Not distinguished from Birch/Coniferous using IKONOS
Other	1.29	8.12	18.51	18.51	Includes: roads, bare ground, tundra, lakes and rivers
(Misc)	(1.29)	(8.12)	(13.44)	(13.44)	Roads, bare ground and tundra (mainly tundra in IKONOS)
(lakes/rivers)	(n/a)	(n/a)	(5.07)	(5.07)	Lakes and rivers

Due to the resolution and spatial extent of the data, classifications from the aerial photography and IKONOS differ; shading indicates which aerial photography classifications should relate to each IKONOS classification.

### Upscaling to the main 100 km^2^ area

The IKONOS satellite data for the full 100 km^2^ area were classified into five main landscape units: mire, deciduous forest, evergreen forest, tundra and bare rock, and lakes. Two‐thirds of the area was classified as deciduous (birch) forest, with mires covering ~ 11 % of the landscape (Fig. 5; Table 2). The strong agreement between the eddy covariance data and the upscaled chamber fluxes, as well as the ability of the satellite data to identify the spatial extent of graminoid lawns, allowed for upscaling to a larger geographical area. The total amount of CH_4_ emitted by the mires was much greater than the amount of CH_4_ consumed by the birch forest (Table 3). Uncertainty caused by differences between the aerial photography and satellite vegetation classification was taken into account and included in the calculation of the CH_4_ fluxes.

**Table 3:** The influence of uncertainty in the vegetation classification on calculated landscape‐level CH_4_ fluxes. Average flux rates between 1st May and 31st October are presented, together with cumulative amounts of CH_4_ emitted over the full 6 month period

Classification	Emission		Uptake		Net flux		Uptake (%)
	Average flux (nmol m^−2^ s^−1^)	Cumulative (g CH_4_ m^−2^)	Average flux (nmol m^−2^ s^−1^)	Cumulative (g CH_4_ m^−2^)	Average flux (nmol m^−2^ s^−1^)	Cumulative (g CH_4_ m^−2^)	
Raw ex. conifer and tundra	5.866	1.492	0.468	0.119	5.398	1.373	7.976
Raw inc. conifer and tundra^a^	5.866	1.492	0.598	0.152	5.268	1.340	10.188
Adjusted ex. conifer and tundra	4.871	1.239	0.279	0.071	4.592	1.168	5.730
Adjusted Inc., Conifer and tundra^a^	4.871	1.239	0.405	0.103	4.466	1.136	8.313

a Fluxes were calculated from both the raw and adjusted satellite classification (see Table 2), and either include or exclude potential methane uptake by coniferous forest and tundra soils, the two ecosystem types in which no flux measurements were made.

We calculate that the birch forest consumed 6–10% of the CH_4_ being emitted by the graminoid lawns, and either excluding or including the tundra and coniferous forests as CH_4_ consuming ecosystems had little impact on the calculated flux rates (Table 3). Reflecting the dominance of the lawn emissions, flux rates at the landscape level were greatest during the warmest period of the year (Table 4). Over the full season, in our 100 km^2^ study area, the average cumulative amount of CH_4_ emitted between 1st May and 31st October was calculated to be between 1.1 and 1.4 g m^−2^, depending on the vegetation classification applied (Table 3).

**Table 4:** Mean monthly fluxes from the lawns and calculated for the full 100 km^2^ area

	Average methane flux (nmol m^−2^ s^−1^)	
Month	Lawns	Landscape^a^
May	24.89	2.20
June	62.96	5.70
July	86.42	7.85
August	62.18	5.63
September	37.40	3.36
October	24.82	2.20

a Landscape‐level fluxes are based on modelling the chamber measurements using the adjusted classification, including potential uptake by conifer and tundra soils (see Tables 2, 3).

## Discussion

### Spatial variability in CH_4_ fluxes

The sedge‐dominated lawns were the largest sources of CH_4_ in the aapa mire (Fig. 2), with these emissions found to be the dominant CH_4_ flux at the landscape scale. The wettest, *Sphagnum*‐dominated interhummocks also emitted substantial quantities of CH_4_, but flux rates from the other mire plant communities were an order of magnitude lower. Average CH_4_ emission rates in the lawns during the middle of the growing season were high (~ 90 nmol m^−2^ s^−1^; Fig. 2 and Table 4), but in close agreement with fluxes measured in other tall sedge‐dominated wetlands in Fennoscandia, including the ~ 100 nmol m^−2 ^s^−1^ measured in another aapa mire in northern Finland (Heikkinen *et al*., 2002), the ~ 110 nmol m^−2 ^s^−1^ observed in a subarctic palsa mire in northern Sweden (Jackowicz‐Korczyński *et al*., 2010), and the ~ 70 nmol m^−2^ s^−1^ measured in a sedge‐dominated fen in southern Finland (Rinne *et al*., 2007).

In contrast, net CH_4_ uptake was observed in the birch forest soils, with rates being broadly comparable to those observed in other boreal forests (Dutaur & Verchot, 2007; Matson *et al*., 2009). However, maximal rates of uptake were found to be ~ 100 times lower than the rate of CH_4_ emission measured in the lawns during the middle of the growing season. Overall, CH_4_ flux rates were extremely variable spatially, and thus detailed information on the distribution of the different plant communities and mire subunits was required for upscaling.

### Environmental control over CH_4_ fluxes

CH_4_ emissions from the mire lawns and *Sphagnum*‐dominated interhummocks were found to be positively related to seasonal changes in temperature, while there was also a positive relationship (*P* = 0.098, *R* ^2^ = 0.420) between temperature and the rate of CH_4_ consumption in the birch forest (Fig. 3, Table 1). In contrast, CH_4_ fluxes in the other plant communities were not related to temperature, but were related to water‐table depth and soil moisture on the lichen heath. Our results are broadly in agreement with the reviews by Olefeldt *et al*. (2013) and Turetsky *et al*. (2014), which also observed that temperature was the best predictor of temporal variation in CH_4_ fluxes in the wettest parts of the landscape, but that water‐table depth was a better predictor of emission rates in slightly drier ecosystems.

There are challenges associated with separating the direct effects of seasonal changes in temperature, from mid‐season peaks in plant productivity. Christensen *et al*. (2003) demonstrated that acetate concentrations in pore water could explain over 90% of spatial variation in CH_4_ fluxes across a range of high‐latitude wetlands, demonstrating the importance of substrate availability. Higher temperatures may increase overall rates of decomposition and thus increase acetate concentrations, but may also promote greater plant productivity and thus increase root turnover and exudation (Qian *et al*., 2010; Epstein *et al*., 2012). For the two major CH_4_ emitting plant communities, the floating nature of the vegetation in the sedge‐dominated lawns means that a very substantial increase in the water table depth (>1 m in the lawns) would be required before these subunits would start to dry out. Therefore, CH_4_ fluxes from aapa mires in northern Finland are likely to increase in the future if the climate continues to warm, due both to the direct effects of rising temperatures on decomposition rates, and the potential for increases in plant growth and the length of the growing season.

### Intercomparison between chamber and eddy covariance data

In agreement with previous studies (Riutta *et al*., 2007; Hendriks *et al*., 2010; Parmentier *et al*., 2011), we observed good agreement between the eddy covariance measurements and fluxes upscaled from chamber measurements using relationships with the environmental variables, the aerial photography vegetation classification and eddy tower footprint modelling (*R* ^2^ = 0.72; Fig. 6b). This even extended to a three‐day period in late August, during which the footprint model indicated that there were large fluctuations in the contribution of CH_4_ emitting vs. consuming vegetation communities to the eddy covariance fluxes. These fluctuations were reflected in both the timing and magnitude of the changes in fluxes calculated by the two methods (Fig. 4b, Fig. S1). This suggests that potential methodological problems associated with chamber flux measurements (Denmead, 2008) were not substantial at our field site, and, thus, allowed us to up‐scale the chamber fluxes to the wider landscape with confidence.

### Estimating landscape‐level fluxes

We found that the IKONOS data were able to detect the wettest areas of the mires very well, but not the hummocks and interhummocks. This has minimal impact in terms of the upscaled CH_4_ fluxes because the areas of mire detected clearly in the satellite classification correspond to the key CH_4_ emitting plant communities (Fig. 5). Indeed, the fact that the satellite identified the area of lawns rather than the total mire area actually reduced uncertainty in the upscaling. This is because the mire lawn fluxes were much greater than the fluxes from all other vegetation communities. For this reason, upscaling the chamber flux measurements, using the raw IKONOS classification, already showed good agreement with both the eddy covariance data and the chamber flux measurements upscaled using aerial photography (Fig. S5). On the other hand, if the satellite classification had identified the total area of mire, we would have had to estimate the total area of lawn adding considerable uncertainty to the calculation. We only had to use the aerial photography/IKONOS intercomparison to estimate the coverage of vegetation communities that were not major CH_4_ emitters.

In many boreal and subarctic regions, graminoid lawns are the key CH_4‐_producing plant communities (Heikkinen *et al*., 2002; Jackowicz‐Korczyński *et al*., 2010), and thus, the utility of widely available satellite data for detecting this key community is potentially very significant. As well as its utility in upscaling, we suggest that the ability of the satellite data to identify the spatial coverage of lawns also has potential for measuring changes in the area of CH_4_ emitting communities where there is ongoing permafrost thaw. The collapse of permafrost plateaus in northern boreal and subarctic wetlands often results in water logging with lichen and shrub‐dominated communities being replaced by graminoid and *Sphagnum* lawns (Turetsky *et al*., 2007; Jackowicz‐Korczyński *et al*., 2010; Johnston *et al*., 2014). Thus, there is also potential for using satellite data of equivalent spatial resolution (5 m or better) in this context to improve predictions of changes in landscape‐level CH_4_ fluxes in permafrost regions.

It has been suggested that the areal extent of CH_4_ emitting subunits will be increasingly underestimated as the spatial resolution of the remote sensing data used declines (Becker *et al*., 2008). However, the area of mire detected in the satellite data here was ~ 20% greater than the area of lawns detected in the aerial photography. This difference added uncertainty to the upscaling of CH_4_ emissions, with lower emissions calculated for the adjusted IKONOS classification after accounting for this potential slight overestimation in lawn extent (Table 3). In addition, lichen heath and mire edge were not distinguishable from birch forest in the IKONOS data due to similarities in their spectral properties, and this contributed to uncertainty in the calculation of total rates of CH_4_ uptake in drier ecosystems; the proportion of lawn emissions being consumed varied from 6 to 10% depending on the classification applied (Table 3). However, in terms of the net CH_4_ flux, the uncertainty in the classification of the area of forest, lichen heath and mire edge did not affect the flux substantially. Finally, the inclusion or exclusion of the unmeasured ecosystems (tundra and coniferous forest), only contributed to an uncertainty in the landscape flux of 0.03 g CH_4_ m^−2^. Put together, the uncertainty in the vegetation classification, resulted in the average amount of CH_4_ released during the growing season being calculated at between 1.1 and 1.4 g CH_4 _m^−2^ (Table 3), not including potential fluxes from lakes.

For comparison, in the Torneträsk catchment in northern Sweden, fluxes at the landscape level were calculated at only ~ 0.25 g CH_4_ m^−2 ^year^−1^, again when fluxes from lakes were excluded (Christensen *et al*., 2007). Furthermore, it was estimated that greater than 25% of the CH_4_ released from wetlands was potentially consumed in the more freely drained systems in the Swedish study. This was due to the greater proportion of relatively well‐drained hill slopes and smaller area of poorly drained lowlands compared with our study area. In addition, while Christensen *et al*. (2007) used the Swedish CORINE Land‐Cover Map, here, the higher, 4 × 4 m resolution of the IKONOS classification was very important in detecting the area of small lawns (Fig 5). Overall, the presence of more spatially extensive aapa mire systems in northern Finland resulted in landscape‐level CH_4_ fluxes being 4–6 times greater than those measured in northern Sweden (Christensen *et al*., 2007).

Greenhouse gas emissions from lakes are receiving increasing attention. Previous studies have suggested that CH_4_ fluxes from high‐latitude lakes, especially small lakes, may be greater per unit area than fluxes from some mires [e.g. >20 g CH_4_ m^−2^ yr^−1^ for lakes above 66^o^N (Bastviken *et al*., 2011)]. However, this figure was derived mainly from permafrost thaw lakes, and fluxes from lakes in Fennoscandia appear to be lower [0.2–14 g m^−2^ year^−1^; (Bastviken *et al*., 2011; Juutinen *et al*., 2009)], although ebullition fluxes were not always measured in these data sets. Given that lakes were not included in the current study, fluxes at the landscape level may have been underestimated by ~ 0.35 g CH_4_ m^−2^ year^−1^ or 25–30%. This estimate is based on assuming a flux rate of 7 g CH_4_ m^−2^ year^−1^, the mid‐point of the range of flux rates for Fennoscandian lakes (Juutinen *et al*., 2009; Bastviken *et al*., 2011).

Although our landscape‐level fluxes are high in a Fennoscandian context, average mid‐season, landscape‐level fluxes measured in areas of Siberia are considerably higher. In our study, mid‐season fluxes were ~ 10 nmol m^−2^ s^−1^, but across a 400 by 400 km^2^ area in the west Siberian lowlands they were calculated to be greater than 40 nmol m^−2^ s^−1^ (Takeuchi *et al*., 2003; Sasakawa *et al*., 2012). A larger proportion of mire in the landscape and a greater flux rate per unit mire area, contributed equally to the greater fluxes calculated in these studies, compared with our analysis. The greater flux per unit area may have been related to the Siberian sites being considerably further south, and thus warmer during summer months. This further emphasises the potential for substantial increases in CH_4_ emissions in Fennoscandia as temperatures rise at high latitudes.

In summary, in combining chamber measurements, eddy covariance, aerial photography and satellite remote sensing, we have been successful in scaling up CH_4_ fluxes to a 100 km^2^ area of northern Finland. Using this novel approach, we conclude that the graminoid lawns present in the aapa mires in this region dominate the landscape‐level CH_4_ flux. Soils in the more free‐draining areas of the landscape were likely capable of consuming only a small proportion (10% or less) of the CH_4_ being released. Fluxes at the landscape level were found to be high in a Fennoscandian context, with on average 1.1–1.4 g CH_4_ m^−2^ being released over the course of the growing season, not including lake fluxes. Finally, the strong relationship between temperature and rates of CH_4_ release suggests that CH_4_ emissions from aapa mire regions will increase rapidly in the future if temperatures rise and plant productivity increases.

## Supporting information


**Fig S1.** Example of how the flux footprint shifted during the course of a single day (26th August 2008). The 48 half‐hourly flux footprints calculated using the Kormann & Meixner (2001) model are shown.
**Fig S2.** Relationships between the proportion of graminoid lawns calculated to be within the eddy covariance flux footprint, and the CH_4_ flux (a) across all three campaigns and (b, c, d) for each campaign individually. Significant positive relationships were observed for the full dataset (*P* < 0.001, *R* ^2^ = 0.047) and for Campaigns 2 (*P* < 0.001, *R* ^2^ = 0.086) and 3 (*P* < 0.001, *R* ^2^ = 0.212).
**Fig S3.** Landcover classifications based on the IKONOS satellite data for the full 100 km^2^ area. Coordinates are shown in UTM (zone 35N).
**Fig S4.** The crop of the satellite classification corresponding to the area covered by the aerial photography (a) and the classification based on the aerial photography survey is shown in panel (b). Coordinates are shown in UTM (zone 35N). In the aerial photography, the location of the eddy covariance tower is indicated with a star.
**Fig S5.** CH_4_ fluxes measured using eddy covariance and the modelled fluxes based on up‐scaling the chamber fluxes to the eddy covariance footprint. Fluxes were up‐scaled using both the aerial photography and the raw IKONOS classification (see Table 2; Fig. 5).
