# Supplementary material for: Quantifying landscape‐level methane fluxes in subarctic Finland using a multiscale approach
Source: Glob Chang Biol. 2015 Jun 27;21(10):3712–25. doi: 10.1111/gcb.12975 (PMC4989475; doi:10.1111/gcb.12975)
Supplement: Supplementary file 1 — Fig S1. Example of how the flux footprint shifted during the course of a single day (26th August 2008). The 48 half‐hourly flux footprints calculated using the Kormann & Meixner (2001) model are shown. Fig S2. Relationships between the proportion of graminoid lawns calculated to be within the eddy covariance flux footprint, and the CH4 flux (a) across all three campaigns and (b, c, d) for each campaign individually. Significant positive relationships were observed for the full dataset (P < 0.001, R 2 = 0.047) and for Campaigns 2 (P < 0.001, R 2 = 0.086) and 3 (P < 0.001, R 2 = 0.212). Fig S3. Landcover classifications based on the IKONOS satellite data for the full 100 km2 area. Coordinates are shown in UTM (zone 35N). Fig S4. The crop of the satellite classification corresponding to the area covered by the aerial photography (a) and the classification based on the aerial photography survey is shown in panel (b). Coordinates are shown in UTM (zone 35N). In the aerial photography, the location of the eddy covariance tower is indicated with a star. Fig S5. CH4 fluxes measured using eddy covariance and the modelled fluxes based on up‐scaling the chamber fluxes to the eddy covariance footprint. Fluxes were up‐scaled using both the aerial photography and the raw IKONOS classification (see Table 2; Fig. 5). [file GCB-21-3712-s001.doc]

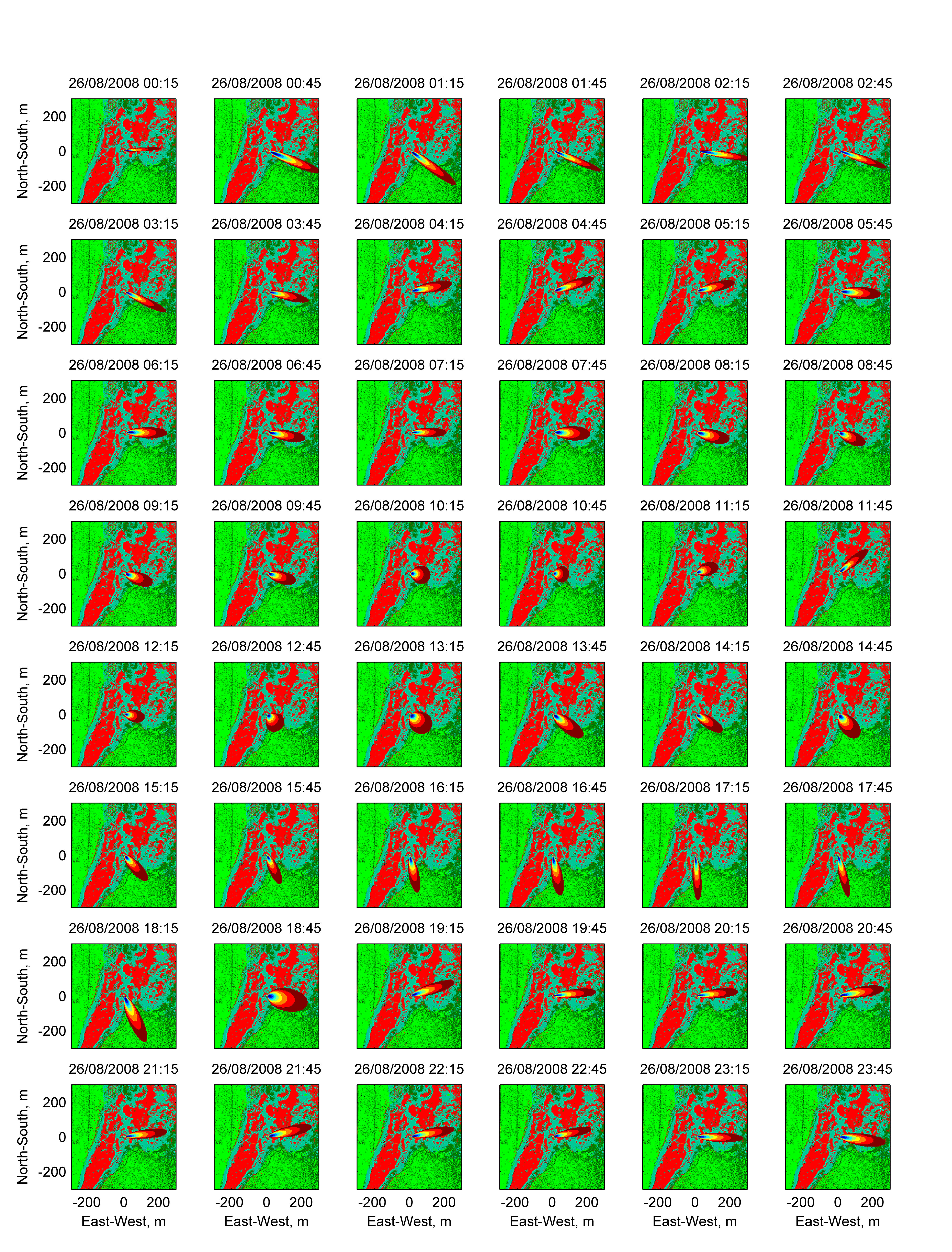


**Fig S1.** Example of how the flux footprint shifted during the course of a single day (26^th^ August 2008). The 48 half-hourly flux footprints calculated using the Kormann and Meixner (2001) model are shown.

**Fig S2.** Relationships between the proportion of graminoid lawns calculated to be within the eddy covariance flux footprint, and the CH_4_ flux (a) across all three campaigns and (b,c,d) for each campaign individually. Significant positive relationships were observed for the full dataset (P<0.001, R^2^ = 0.047) and for Campaigns 2 (P<0.001, R^2^ = 0.086) and 3 (P<0.001, R^2^ = 0.212).

**
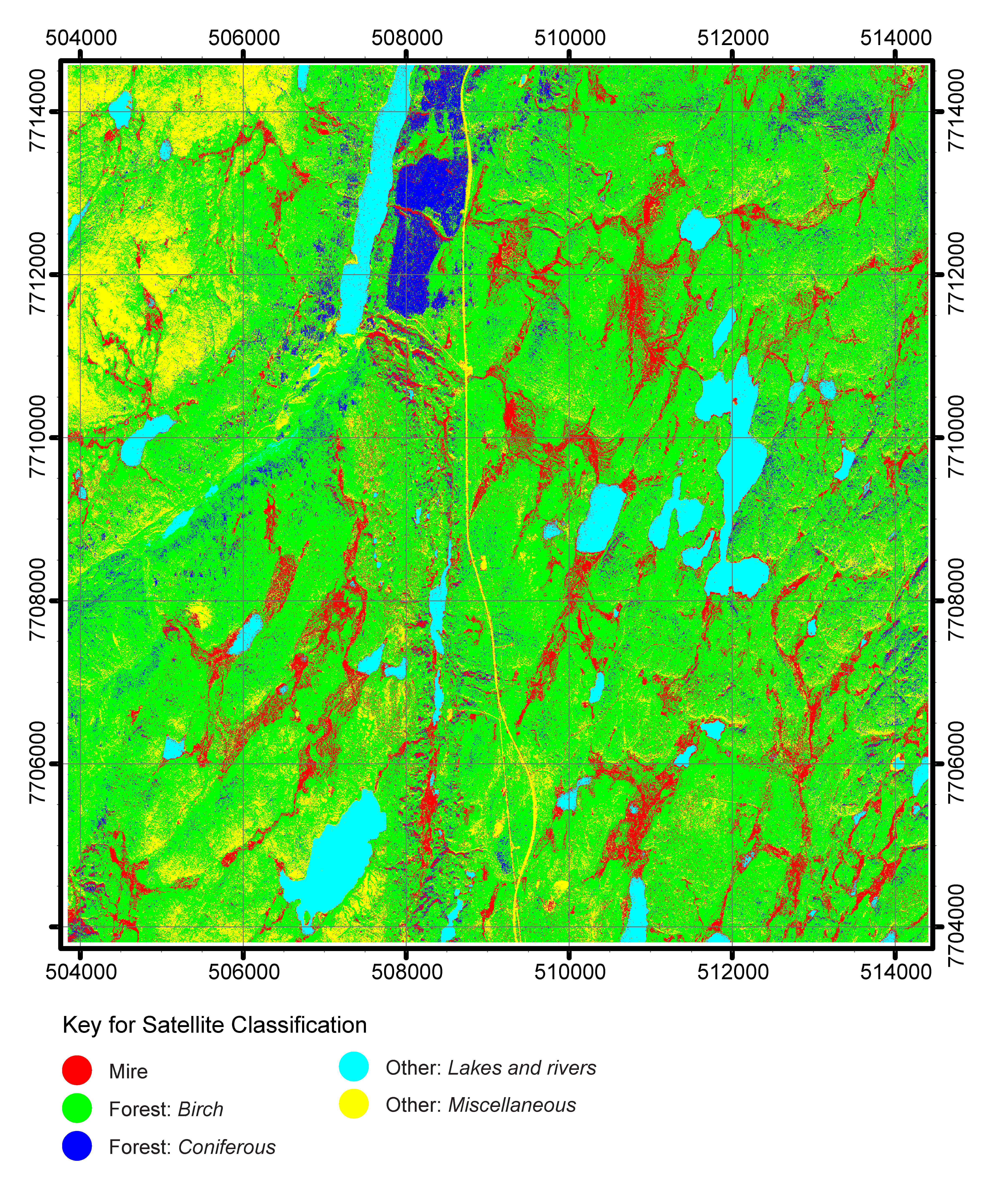
**

**Fig S3.** Landcover classifications based on the IKONOS satellite data for the full 100 km^2^ area. Coordinates are shown in UTM (zone 35N).

**
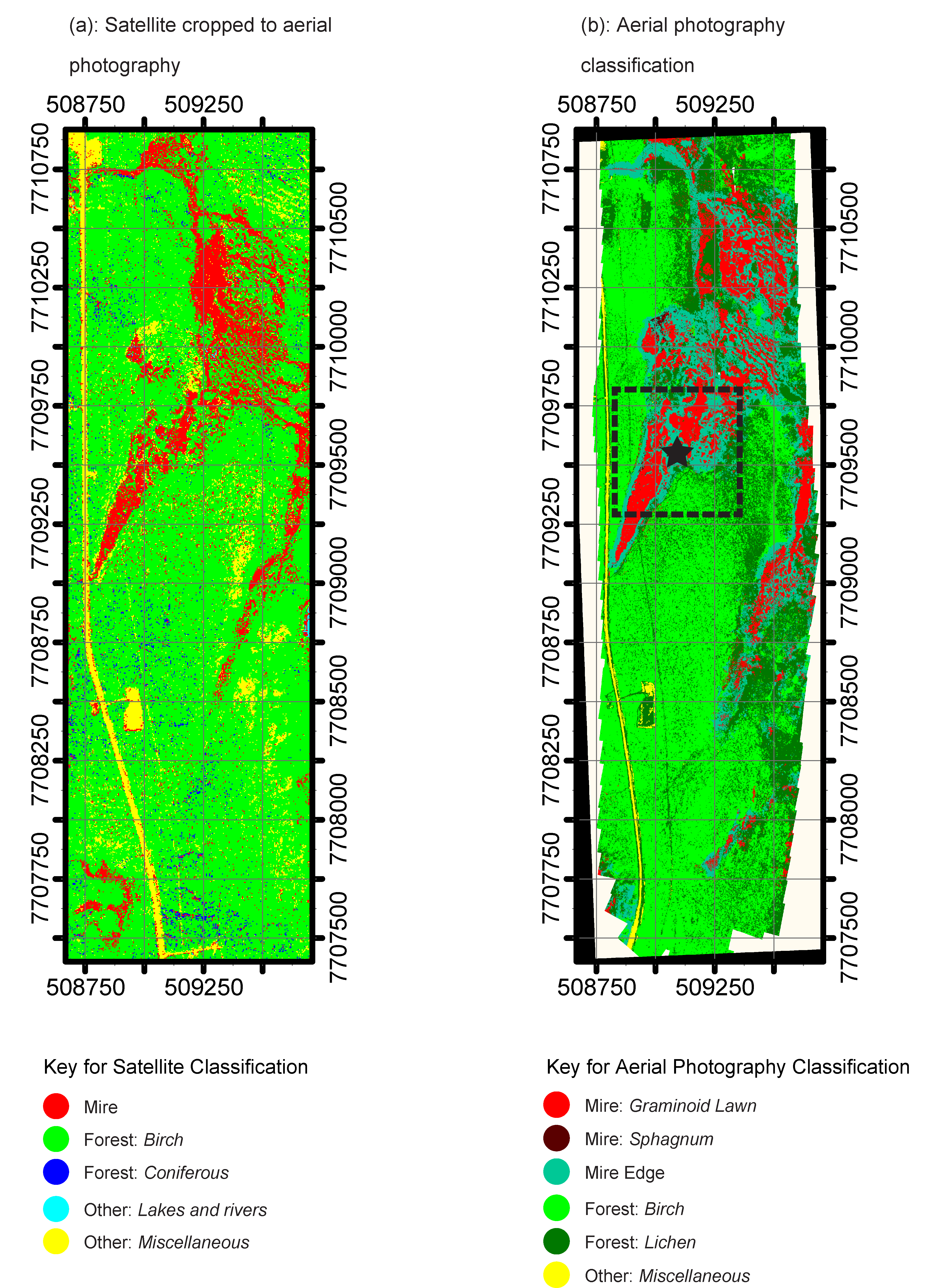
**

**Fig S4.** The crop of the satellite classification corresponding to the area covered by the aerial photography (a) and the classification based on the aerial photography survey is shown in panel (b). Coordinates are shown in UTM (zone 35N). In the aerial photography, the location of the eddy covariance tower is indicated with a ★.

**Fig S5.** CH_4_ fluxes measured using eddy covariance and the modelled fluxes based on up-scaling the chamber fluxes to the eddy covariance footprint. Fluxes were up-scaled using both the aerial photography and the raw IKONOS classification (see Table 2; Fig. 5).
